# Temporary cognitive impairment related to administration of newly developed anticholinergic medicines for overactive bladder: two case reports

**DOI:** 10.1186/1756-0500-7-672

**Published:** 2014-09-25

**Authors:** Takako Shiota, Kazumasa Torimoto, Hitoshi Momose, Takuya Nakamuro, Hiroshi Mochizuki, Hiromi Kumamoto, Akihide Hirayama, Kiyohide Fujimoto

**Affiliations:** Department of Urology, Saiseikai Nara Hospital, 4-643 Hachijo, Nara-shi, Nara, Japan; Department of Urology, Nara Medical University, 840 Shijo-cho, Kashihara-shi, Nara, Japan; Department of Urology, Hoshigaoka Medical Center, 4-8-1 Hoshigaoka, Hirakata-shi, Osaka, Japan; Department of Neurology, Saiseikai Nara Hospital, 4-643 Hachijo, Nara-shi, Nara, Japan

**Keywords:** Overactive bladder, Anticholinergic medicine, Cognition

## Abstract

**Background:**

Cognitive impairment is one of the side effects of using anticholinergic medicines for overactive bladder; however, its incidence has not been fully reported. We experienced two elderly Japanese patients with overactive bladder who had temporary cognitive impairment caused by anticholinergic medicines.

**Case presentation:**

The first case was a 79-year-old female patient to whom imidafenacin (0.2 mg) was administered daily to control her frequent micturition and urgency. She was taking the following medicines: etizolam, triazolam, captopril, bisoprolol, and amlodipine besylate. Her Hasegawa dementia rating scale-revised was impaired from 26/30 to 17/30 and recovered to 25/30 after the imidafenacin treatment was stopped. The second case was an 82-year-old female patient to whom imidafenacin (0.2 mg) was administered daily for frequent micturition and urgency. She was taking the following medicines: losartan potassium and clenbuterol. Her Hasegawa dementia rating scale-revised decreased from 28/30 to 19/30 and recovered to 24/30 after the imidafenacin treatment was stopped. In our patients who were taking multiple medicines, there is a possibility that medicines other than anticholinergics may have caused cognitive impairment. We need to keep in mind that many elderly people take multiple medicines because of comorbidity.

**Conclusions:**

Anticholinergic medicines can cause cognitive impairment in elderly people, and attention should be paid to cognition when elderly overactive bladder patients are treated with anticholinergic medicines.

## Background

Cognitive impairment is one of the side effects of using anticholinergic medicines for overactive bladder (OAB); however, its incidence has not been fully reported. Oxybutynin has been shown to cause cognitive impairment and delirium in elderly people [1]. However, no other report of cognitive impairment caused by anticholinergic medicines for OAB was found. Recently, newly developed anticholinergic medicines for OAB were found to not affect cognition [2, 3]. These newly developed anticholinergic medicines for OAB are thought to be safe and not cause cognitive impairment because their movement into the central nervous system is limited [4]. We experienced two elderly Japanese patients with OAB who had temporary cognitive impairment caused by anticholinergic medicines.

## Case presentation

### Case 1

A 79-year-old female patient presented to a neurologist with a chief complaint of amnesia six months ago and was found to have normal cognition, as a computed tomography (CT) scan did not detect any organic brain lesions and Hasegawa dementia rating scale-revised (HDS-R) [5] was 26/30. She was taking the following medicines: etizolam, triazolam, captopril, bisoprolol, and amlodipine besylate.

**Figure 1:**
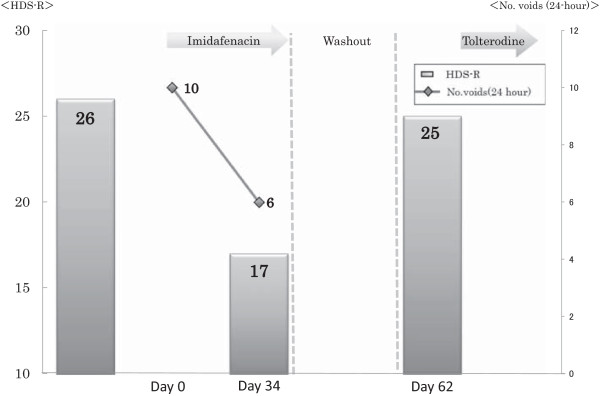
**Monitoring of cognitive function in Case 1.** In Case 1, Hasegawa dementia rating scale-revised score decreased after imidafenacin administration and recovered after the stop of imidafenacin. Bar graph: Hasegawa dementia rating scale-revised, Line graph: 24-hour number of voids.

Imidafenacin (0.2 mg) was administered daily to control her frequent micturition and urgency. After one month, the imidafenacin treatment was stopped because her cognition was impaired (HDS-R, 17/30), although the lower urinary tract symptoms (LUTS) improved. A CT scan did not detect any brain lesions at that time. After three weeks, her cognition recovered (HDS-R, 25/30). Then, tolterodine (4 mg) was administered daily instead of imidafenacin; however, it was stopped because of cognitive impairment (Figure 1).

### Case 2

**Figure 2:**
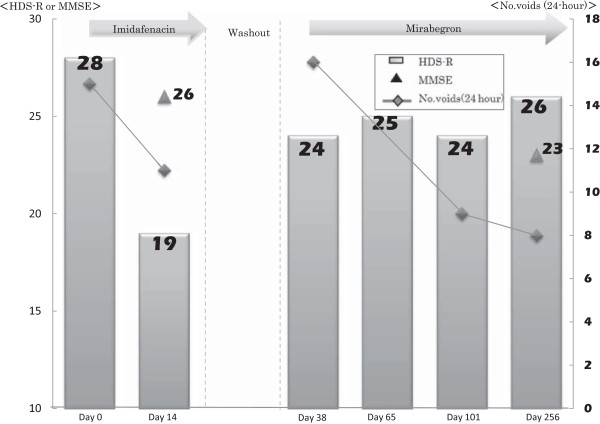
**Monitoring of cognitive function in Case 2.** In Case 2, Hasegawa dementia rating scale-revised decreased after imidafenacin administration and recovered after the stop of imidafenacin although mini mental scale examination was stable. Bar graph: Hasegawa dementia rating scale-revised, Line graph: 24-hour number of voids, Filled triangle: mini mental scale examination.

An 82-year-old female patient presented with a chief complaint of mixed urinary incontinence that had persisted for four months. She was taking losartan potassium. Clenbuterol (40 μg) was administered daily because stress urinary incontinence was predominant over urge urinary incontinence. After one month, her urinary incontinence improved. Three months later, imidafenacin (0.2 mg) was administered daily for frequent micturition and urgency. However, after two weeks of treatment, her HDS-R decreased from 28/30 to 19/30, although the LUTS improved. At that time, she was found to have normal cognition by a neurologist because her mini mental scale examination (MMSE) was 26/30 and a CT scan did not detect any brain lesions. Imidafenacin administration was immediately stopped, and three weeks later, her HDS-R recovered to 24/30, although the LUTS got worse. Then, mirabegron (50 mg) was administered daily instead of imidafenacin. Her cognition was stable as follows: her HDS-R was 25/30 after one month, and her HDS-R and MMSE were 26/30 and 23/30, respectively, after seven months (Figure 2).

## Discussion

Anticholinergics are the first choice for OAB and are often administered to elderly people in whom the prevalence of OAB is high [6]. While it has been reported that anticholinergic medicines for OAB are safe and do not cause cognitive impairment [4], anticholinergics do in fact frequently induce reversible cognitive impairment [7, 8]. They were traditionally thought to affect cognition and be high-risk medicines causing cognitive impairment. There is good evidence that the combination of multiple medicines can precipitate or predispose to cognitive impairment. The American College of Surgeons National Surgical Quality Improvement Program (ACS NSQIP®)/American Geriatrics Society (AGS) Best Practices Guidelines list strong anticholinergic medicines for OAB, such as darifenacin, fesoterodine, flavoxate, oxybutynin, solifenacin, tolterodine, and trospium, which may be avoided in elderly people [9]. The adverse effects of anticholinergic medicines on cognition depend on the strength or combination. It has been reported that anticholinergic medicines had a significantly high risk of causing cognitive impairment and dementia after adjusting for other possible determinants of cognitive impairment [7, 10]. In our patients who were taking multiple medicines, there is a possibility that medicines other than anticholinergics may have caused cognitive impairment. We need to keep in mind that many elderly people take multiple medicines because of comorbidity.

Cystometry has become unnecessary to diagnose patients as OAB since the Standardization Subcommittee of the International Continence Society (ICS) recognized OAB as a “symptom syndrome suggestive of lower urinary tract dysfunction” which is specifically defined as “urgency, with or without urge incontinence, usually with frequency and nocturia” in 2002 [11]. Afterwards any medical doctors including primary care doctors can diagnose patients as OAB and treat OAB mainly with anticholinergic medicines. We may have to perform cystometry more aggressively to detect detrusor overactivity in elderly patients who have the risk of drug-induced cognitive impairment. That may result in better selection of elderly patients and allow doctors to avoid side effects of anticholinergic medicines.

While MMSE is the global standard diagnostic method for cognition, HDS-R is the standard method in Japan. In our second case (Case 2), the diagnostic results were different between MMSE and HDS-R. It is still debatable which method is better for the diagnosis of cognitive impairment [5].

## Conclusions

Anticholinergic medicines can cause cognitive impairment in elderly people, and attention should be paid to cognition when elderly OAB patients are treated with anticholinergic medicines.

## Consent

Written informed consent was obtained from the patients for publication of these Case Reports and any accompanying images. Copies of the written consent are available for review by the Editor-in-Chief of this journal.

## Abbreviations

OAB: Overactive bladder
HDS-R: Hasegawa dementia rating scale-revised
CT: Computed tomography
LUTS: Lower urinary tract symptoms
MMSE: Mini mental scale examination.
